# U.S. Food and Drug Administration: Review for the Emergency Physician of Approval Process and Limitations

**DOI:** 10.5811/westjem.2016.8.31197

**Published:** 2016-09-12

**Authors:** Nadia Zuabi, Bhavesh Patel, Mark I. Langdorf

**Affiliations:** * University of California, Irvine, School of Medicine, Irvine, California; † University of California, Irvine, School of Biological Sciences, Irvine, California; ‡ University of California, Irvine, School of Medicine, Department of Emergency Medicine, Irvine, California

## INTRODUCTION

Emergency physicians (EP) frequently are exposed to promotion for drugs and devices through professional organizations and meetings, journals, and direct-to-consumer pharmaceutical advertising (DTCPA). To provide optimum patient care through evidence-based medicine, it is critical to be aware of the processes that regulate these drugs.

Though it is uncommon for ED patients to request specific drugs or treatments for emergency conditions, it is not uncommon for patients taking newly marketed drugs with unfamiliar mechanisms of action and side effects to present to the ED. The U.S. Food and Drug Administration (FDA) rate of approval of new drugs is increasing moderately, from 22 in 2006, to 45 in 2015.^1^ This requires the prudent EP to query drug databases for interactions with standard ED treatments, or run the risk of new interactions. Furthermore, nonspecific symptoms may be side effects of new medications, with which the practicing EP is unfamiliar.

The FDA is responsible for strictly regulating the safety and effectiveness of drugs produced by the pharmaceutical industry. The FDA has experienced increasing pressure to fulfill this regulatory role despite the increasing pace of development of medical devices and medications^2^ and a budget that is a fraction of other government agencies. For example, the FDA has only 1.8% of the U.S. Department of Agriculture’s budget.^3^,^4^ This balancing of public health protection and efficiency led Congress to pass the Prescription Drug User Fee Act (PDUFA)^5^ that enabled direct pharmaceutical company subsidization of the FDA review process. The regulatory agency is partially funded by the companies it is charged with regulating. In addition, although the FDA relies on congressional oversight to safeguard 25% of products and services consumed in the U.S.,^6^ robust lobbying influences regulation of these products, which enhances pharmaceutical industry profits.^7^

Despite precautions taken by the FDA, limited funding and external pressure to expedite approval of advanced medical therapies has led to compromises in drug safety. Properly prescribed drugs result in over 100,000 deaths annually, with prescription drugs among the top 10 causes of death, more than each of lung disease, diabetes, AIDS or automobile fatalities.^8^ In 2012 there were approximately 4.2 billion prescriptions written, worth some $326 billion dollars.^9^ Almost 7% of hospitalized patients have a serious adverse drug reaction with a fatality rate of 0.32%.^10^

This paper reviews the FDA’s position in government, limitation of powers and relations with the pharmaceutical industry. These factors have broad influence on the population of patients seeking care in ED.

### Center for Drug Evaluation and Research and Marketing

The Center for Drug Evaluation and Research (CDER) is the branch of the FDA concerned with the review of over-the-counter and prescription drugs.^11^ CDER’s main objective is to evaluate new drugs before they are sold, and provide doctors and patients with information needed to use the medicines wisely. The FDA does not develop, test or manufacture drugs, but instead reviews full reports of clinical studies to determine benefit-to-risk relationship and approval.^12^

Although known as the “consumer watchdog,” concerns of drug safety and timeliness of the FDA review highlight challenges with the current system. This includes an underdeveloped Adverse Effect Reporting System, which is meant to continue surveillance and study of drugs after release in the market, as well as poor enforcement of direct-to-consumer advertising constraints. 12 Title 21 of the Code of Federal Regulations (CFR) is reserved for the FDA and outlines rules published in the Federal Register by executive departments and federal government agencies related to DTCPA.^13^

The need for improved surveillance and study of drugs after approval can be seen with the recent safety labeling changes for fluoroquinolones announced by the FDA in May 2016, when it was reported that the “side effects associated with fluoroquinolones generally outweigh the benefits for patients.” The drug is linked to “disabling and potentially permanent side effects” involving the musculoskeletal and central nervous systems, peripheral neuropathy and cardiovascular complications. Despite these risks and because of challenges associated with post-marketing surveillance, companies such as Bayer, the creator of ciprofloxacin (a type of fluoroquinolone), is still profiting from sales of this drug.^14^,^15^

DTCPA started in 1981. The U.S. and New Zealand are the only countries that allow these advertisements to include product claims.^16^ DTCPA funding from pharmaceutical companies expanded from $791 million in 1996 to $5.4 billion in 2006. The average American television viewer sees nine drug advertisements daily, which equates to about 16 hours per year. This far exceeds the time spent with a primary care physician.^17^

The FDA requires DTCPA to be “fairly balanced” with respect to benefits and risks, to only discuss FDA-approved indications and to explain all possible negative health outcomes whenever the name of the drug is included in the advertisement.^18^ When the FDA believes that an advertisement is misleading, it sends a regulatory letter to the pharmaceutical company. However, since 2002 the FDA has been required to send a draft of the letter to the Department of Health and Human Services for legal review. This substantially increases the time between identifying a violation and notifying the pharmaceutical company. Therefore, many of these letters arrive after the advertisements have already finished airing.^19^

In 2009 59 federal employees were responsible for reviewing 71,759 industry submissions of both DTCPA (radio, television, print, Internet, billboards and direct mailings) and direct-to-physician (DTP) promotional material (detailing brochures that pharmaceutical representatives share with office physicians). As explained above, the FDA can issue a notice of violation through a warning letter when a company violates DTCPA laws. Additionally, it could seek criminal prosecution for repeated violations. However, there are no such known cases.^17^

In November 2015, the American Medical Association (AMA) proposed a ban on DTCPA due to the negative effects on public health and need for transparency on drug pricing. This reflects DTCPA’s role in raising demand for costly drugs despite debate regarding clinical effectiveness in many patients.^16^ However, the DTCPA ban proposed by the AMA is unlikely to be implemented because of the profits gained from off-label use of drugs. For example, Pfizer paid $430 million to settle a claim for fraudulent promotion of the anti-seizure medication Neurontin (gabapentin) when the drug was advertised for non-FDA approved uses such as treatment for neuropathic pain, attention-deficit hyperactivity disorder and as an analgesic for migraine headaches, among others. At the same time, the company made approximately $2.7 billion in sales in a single year, with 90% of the profit from unapproved uses of the drug.^20^ These unapproved uses highlight the consequences of delayed or lax enforcement.

Despite concerns regarding DTCPA, there are studies suggesting that such advertising can be beneficial to patients. There is evidence that DTCPA is a motivating factor for patients to express health concerns to their physician, improve awareness of medical conditions and adhere to prescribed treatments.^21^ A telephone survey of 3,000 adults found that 35% discussed a DTCPA with their physician and 25% of those visits resulted in a new diagnosis.^22^ These findings should be taken into consideration when discussing possible amendments to DTCPA as a promotional tool.

DTCPA drives ED visits and can increase costs, as seen with asthma medications Advair, Asmanex, Singulair and Symbicort,^23^ but has also been shown to improve care specifically in Medicaid-enrolled pediatric patients with asthma.^24^ However, other studies suggest that there are no resulting health benefits from DTCPA.^25^ Low-income patients may be particularly influenced by DTCPA.^26^ As EPs care for a disproportionate share of disadvantaged patients, they need to be aware of the influence of the FDA drug approval process.

### Patents

The Uruguay Rounds Agreements Act (Public Law 103-465) extended the duration of U.S. patents from 17 to 20 years beginning with the date of first filing the patent application.^27^ This gives manufacturers of brand-name drugs sole market rights while in effect. On average, approximately 10 years elapse between the time a patent is obtained and the time the drug is approved, leaving the company about half of the patent time to exclusively market a new drug.^28^ Once the patent expires, 80% of brand-name sales can vanish in a year as generic brands reach the market.^29^

However, in many cases, generic brands can fail to reach the market due to reverse payment patent settlements, or “pay-for-delay” agreements, in which brand-name pharmaceutical companies pay generic competitors to *not* sell cheaper, alternative products. This limiting of competition results in $3.5 billion in higher drug costs every year; restricting these agreements would reduce federal debt by $5 billion over 10 years. 30,31 The conversion of the top 20 drugs from brand-name to generic, in terms of yearly sales and length of delay, was postponed by an average of five years by “pay-for-delay” agreements; and drug companies accrued a combined $98 billion before generic brands were sold. There are reported to be 142 brand-name drugs associated with “pay-for-delay” deals since 2005.^32^ Because of this, the “pay-for-delay” phenomenon has become a prioritized concern for the Federal Trade Commission in recent years. 30,31

Drug companies can file multiple patents in an attempt to extend drug patent life. When a generic drug is challenged in court, the FDA is required by law to freeze approval for 30 months unless the case is settled before that time. The FDA has no authority to litigate patent infringement law.^33^ Members of Congress often tag patent extensions onto bills that favor companies that have contributed to their campaigns. For example, in 2002 Bayer took advantage of campaign contributions to extend its monopoly on Cipro by six months. Three of the four congressional sponsors who approved the bill were among the leading recipients of pharmaceutical company campaign contributions in previous years. Bayer had spent $3.7 million on lobbying efforts for two years, but was able to make $358 million extra profit due to the patent extension.^34^,^35^

Additionally, drug companies file new patents on drugs that are minimally changed compared to the previous version. For instance the company can change the isomer of the drug or change the delivery system to extend patents. In 2008 chlorofluorocarbon, used in inhalers for medications such as albuterol, were banned due to harmful effects on the ozone.^36^ This mandate forced companies to switch to hydrofluoroalkane (HFA)-compatible valves, elastomers and surfactants, all of which allowed for new patents and dramatically increased prices compared to the previous generic brand. The newer HFA metered-dose inhaler (MDI) jumped in price ($42–54) compared to the previous chlorofluorocarbon MDI ($13–17).^37^ Similarly, a device used to administer ipratroprium is associated with 17 separate patents creating a 58-year patent protection lifetime for this medicine. The concept of “evergreening,” defined by Beall et al. as lengthening exclusivity of a product without demonstrating a comparable therapeutic benefit, incentivizes repetitively amending pharmaceutical devices and directing research funding toward promotion of “patentable ideas” instead of medicinally advantageous products.^38^

It has been argued, however, that the profits made from these drugs through patent extensions are necessary to continue funding further development of life-saving treatments. Ensuring profits is especially important due to increasing research expenses, which by 2000 rose to more than $800 million in pre-approval costs per drug.^39^ One method of promoting patent extension is altering formulas to reduce frequency of use, which improves patient adherence to prescribed medications. An example of this can be seen with new extended-release formulas made for the antidepressant Prozac and diabetes medication Glucophage.^40^ This reinforces the idea that extending market exclusivity can in some cases incentivize innovations that result in improved uses and efficacy of drugs.

### PDUFA and the 21^st^ Century Cures Act

In 1992 Congress passed the Prescription Drug User Fee Act, which enabled pharmaceutical company subsidization of the FDA review process. Before PDUFA was passed, taxpayers alone paid for product reviews through budgets provided by Congress.^41^

Pharmaceutical companies pay an application fee for new drug evaluation, the cost of which has risen from $100,000 in 1993 to $2,374,200 per drug in 2016. Product fees are paid annually for previously approved drugs and devices and have increased from $6,000 in 1993 to $144,450 in 2016. In addition, each approved manufacturing facility is assessed an “establishment fee” annually of $585,200 (in 2016) to further support the FDA budget. 42 PDUFA is, therefore, a crucial source of revenue and disincentivizes Congress to fund the FDA. 43

With this increased external source of funding, the PDUFA has undoubtedly accomplished its goal of shortening approval times. In 1987 the median approval time for a new drug application (NDA) or biologic license application (BLA) was 29 months. This number fell to 17 months within the first two years of PDUFA.^41^ This shortened approval time also influenced the number of new drugs that were first introduced in the U.S. In the 1980s only 2–3% of new drugs came from the U.S. This number jumped to 60% in 1998.^44^ The proportion of drugs reviewed and eventually approved rose from 60% in the early 1990s to 80% by 2000.^45^ In 2000, a *Los Angeles Times* report stated that the FDA felt it was being pressured for not only faster reviews on decisions, but also more drug approvals.^46^

The 21^st^ Century Cures Act, passed in July 2015, sought to further accelerate approval times for new products. Before the Cures Act, approximately one-third of new drugs were approved on a single trial with a median sample size of 760 patients. More than two-thirds of new drugs were approved on studies that lasted six months or less, even though these drugs are designed to be taken for much longer periods of time. The majority of drugs were approved within six to 10 months once FDA review began. The Cures Act now seeks to further shorten this approval time by instructing the FDA to use even “shorter or smaller clinical trials” for devices and to rely on evidence from “clinical experience” including “observational studies, registries and therapeutic use,” instead of randomized controlled trials. The FDA is now depending more on biomarkers and surrogate measures rather than actual clinical end points. The FDA already uses surrogate endpoints in about half of new drug approvals.^47^

Furthermore, medical devices have been criticized for lack of rigor compared to drug evaluations. New laws have redefined evidence to include case studies, registries and articles in the medical literature rather than clinical trials. Although informed consent generally is considered to be of utmost importance in the medical community, a clause in the 21^st^ Century Cures Act adds an exception to informed consent for drug and device trials in which “proposed clinical testing poses no more than minimal risk.” It remains poorly defined who determines this minimal risk.^47^

Despite these challenges, the FDA has made noteworthy accomplishments with drug oversight. Currently, the average FDA review time is 40, 70 and 174 days faster than Japan, Canada and Europe respectively. From 2004–2013, 75% of drugs approved in these countries, in addition to Australia, had already been authorized by the FDA.^48^ Therefore, effective and potentially life-saving drugs may often be first available to patients in the U.S. due to the FDA’s regulatory model.

### Lobbying and the UCS Survey

The top 20 pharmaceutical companies along with their two trade groups – Pharmaceutical Research and Manufacturers of America (PhRMA) and Biotechnology Industry Organization – lobbied on over 1,600 pieces of legislation between 1998 and 2004. From January 2005 to June 2006 the pharmaceutical industry disclosed spending $182 million on federal lobbying and has 1,274 registered lobbyists in Washington D.C.^49^

An example of potential conflict of interest through lobbying can be seen with Wilbert “Billy” Tauzin, who represented Louisiana from 1980 to 2005, and became the chair of the House Committee on Energy and Commerce. He crafted the Medicare Prescription Drug, Improvement and Modernization Act of 2003, which prevented Medicare from negotiating for lower prescription drug costs and banned re-importation of drugs from developed-world countries. After the bill passed, Tauzin announced retirement from Congress and took a job as the CEO and chief lobbyist for PhRMA along with an approximate salary of $2 million annually.^50^

These political pressures may have influenced FDA activities, according to the results of the Union of Concerned Scientists (UCS) survey, published in the *Institute of Science in Society.*^51^ It showed:

- “18.4% claimed they ‘have been asked for non-scientific reasons to inappropriately exclude, or alter, technical information or their conclusions in FDA scientific documents.’
- 17% had been asked ‘to provide incomplete, inaccurate or misleading information to the public, regulated industry, media, or government officials.’
- 40% expressed concern of the consequences if they expressed their concerns regarding public health safety in public.
- 47% think that the FDA routinely provides complete and accurate information to the public
- 61% knew of cases where Department of HHS (Health and Human Services) or FDA appointees inappropriately injected themselves into FDA determinations of actions
- 81% agreed that the public would be better served if the independence and authority of FDA post-market safety systems were strengthened.”

### Institute of Medicine on Safety

The Institute of Medicine (IOM) is a nonprofit organization created by Congress to advise the federal government on health issues. In September 2006, the IOM issued a report on drug safety discussing the FDA and the pharmaceutical industry’s lack of accountability to adequately address public health concerns. These issues were partially attributed to limited resources and a suboptimal organizational culture at CDER, as well as an absence of regulatory authority and leadership.^12^

Several recommendations were made to improve the review process. It was proposed that an FDA commissioner with experience and qualifications to lead a science-based agency be selected for a six-year term. The report also suggested that guidance from the Department of HHS would improve morale, professionalism, transparency and integrity of the system. Separation of FDA finances from pharmaceutical companies was also proposed to avoid potential conflicts of interest during the drug review process. It was also recommended to post at least Phase 2 through Phase 4 clinical trials at www.clinicaltrials.gov along with results regarding effectiveness and safety.^12^

The IOM report supported legislation that would enhance FDA authority through restriction of DTCPA as well as better enforcement of fines, warnings and drug approval withdrawals. It was suggested that there be a mandatory evaluation of drugs five years post approval via efficacy and safety reports submitted by drug sponsors. Finally, to support all of the above modifications, it was proposed that Congress should significantly enhance FDA staff and funding.^12^

### Other Ideas

A 2006 article published in the *New England Journal of Medicine* by Dr. Alastair Wood also developed other solutions to many of the issues faced by the FDA and the drug-approval process. With respect to the absence of long-term safety data and head-to-head comparisons, the article proposes providing an extended period of patent exclusivity for drugs that have Phase 4 commitments completed, demonstrate continued safety or show improvement over the same class of drugs on the market as opposed to “non-inferiority.”^52^

He also recommended an extended period of exclusivity for predefined highly demanded and high-risk drugs that clearly demonstrate a “first in class” status. To solve the issues of surrogate markers not equating to clinically meaningful endpoints, the article proposes limited exclusivity for drugs that have been evaluated using surrogate endpoints and extended exclusivity only to drugs that have produced clinically meaningful outcomes. Finally, the article reinforced the importance of limiting accelerated approval exclusively to life-saving drugs, penalizing pharmaceutical companies who attempt to influence the FDA, rewarding FDA employees for reporting such attempts and encouraging patients to report adverse complications.^52^

## CONCLUSION

The FDA must find a balance between hasty drug approvals and meeting demands of advancements in science and technology. Strengthening the authority of the FDA is vital to maintaining integrity and transparency. This translates to distancing individuals and companies that are being regulated from the review process of medical drugs and devices from which they profit. Perhaps most importantly, it is necessary for Congress to develop a plan to properly fund the FDA so that they have the resources to fulfill their responsibilities of protecting public health and safety. Without these reforms, the “watchdog” function will continue to be inadequate to the task.
